# Harmonisation of biobanking standards in endometrial cancer research

**DOI:** 10.1038/bjc.2017.194

**Published:** 2017-06-29

**Authors:** M Adishesh, A Fyson, S B DeCruze, J Kirwan, ENITEC Consortium, H M J Werner, D K Hapangama

**Affiliations:** 1 Liverpool Women’s Hospital NHS Foundation Trust, Crown Street, Liverpool L8 7SS, UK; 2 Department of Women’s and Children’s Health, Institute of Translational Medicine, University of Liverpool, Liverpool Women’s Hospital, Crown Street, Liverpool L8 7SS, UK; 3 Department of Obstetrics and Gynaecology, Haukeland University Hospital, Jonas Liesvei 70, 50503 Bergen, Norway; 4 Department of Clinical Science, University of Bergen, Bergen 5021, Norway

**Keywords:** endometrial cancer, standardisation, harmonisation, biospecimens, data collection tools

## Abstract

**Background:**

Endometrial cancer is the most common gynaecological cancer and its incidence is predicted to escalate by 50–100% in 2025 with a parallel increase in associated mortality. Variations in the collection, processing and storage of biospecimens can affect the generalisability of the scientific data. We aimed to harmonise the collection of biospecimens, clinical data relevant to endometrial cancer and to develop standard operative procedures for the collection, processing and storage of endometrial cancer biospecimens.

**Methods:**

We designed research tools, which were evaluated and revised through three consensus rounds – to obtain local/regional, national and European consensus. Modified final tools were disseminated to a panel (*n*=40) representing all stakeholders in endometrial cancer research for consensus generation.

**Results:**

The final consensus demonstrated unanimous agreement with the minimal surgical and patient data collection tools. A high level of agreement was also observed for the other remaining standard tools.

**Conclusions:**

We here present the final versions of the tools, which are freely available and easily accessible to all endometrial cancer researchers. We believe that these tools will facilitate rapid progress in endometrial cancer research, both in future collaborations and in large-scale multicentre studies.

Endometrial cancer (EC) is the most common cancer of the female genital tract in the developed world, and is the fourth most common cancer in women after breast, lung and colorectal cancer (Ferlay *et al*, 2015). In the United Kingdom in 2014, at least 6 women died of and 21 women were diagnosed with EC in the United Kingdom every day, with 9022 new cases and 2166 deaths reported that year (CRUK). The incidence rate of EC is increasing rapidly and is estimated to increase by 50–100% by 2025 (Lindemann *et al*, 2010). This increase in incidence is alarming, particularly due to the corresponding rise in mortality (CRUK). Increased efforts into finding new prevention, diagnostic, prognostic and therapeutic targets are therefore urgently required to reduce the high mortality and morbidity rates associated with EC. Traditionally, among others immunohistochemistry was used, based on formalin-fixed paraffin-embedded tissue, allowing only for the study of a limited number of proteins simultaneously. Further cell lines and animal studies have been applied in EC research; these however rarely give a perfect simulation of the *in vivo* human environment. Therefore, biobanks, collecting a wide range of different patient specimens, including for example fresh frozen tissue, urine, blood or saliva, have a vital role in providing valuable patient material for clinically relevant scientific discoveries and also aid to the rapid translation of basic scientific findings to clinical practice.

Through its nature, patient material stored in biobanks allows for studying multiple aspects of EC. This is of paramount importance with the emergence of novel technological platforms in genomics, proteomics, epigenomics and metabolomics that can be collectively and simultaneously applied to the same patient samples to gain the maximum amount of information. Such an all-encompassing approach is expected to reduce considerably the time taken for new basic scientific discoveries to reach patients as new treatments as well as allowing the samples donated by patients to be fully used.

The internal and external validity of the generated data depend on their quality, which is clearly dependent on the use of stringent standards in collecting the biospecimens and patient characteristics. Variations associated with collecting, processing, storing different biospecimens and the accompanying phenotypic and demographic data make it extremely difficult to extrapolate or to merge data from different studies (Tworoger and Hankinson, 2006; Ransohoff and Gourlay, 2010). This lack of quality standards and uniformity is recognised by the National Cancer Institute (NCI) as a roadblock in cancer research (NCI Best Practices for Biospecimen Resources, 2011). The irrevocable bias introduced by the irregularities and dissimilarities in specimens and data collection are well recognised by many and efforts are being made to overcome this by several international organisations and agencies (Morente *et al*, 2007; International Society for Biological and Environmental Repositories, 2008; Yuille *et al*, 2008; Vaught and Lockhart, 2012).

The NCI best practice guidance for biobanks (NCI Best Practices for Biospecimen Resources, 2011, 2016), which encourages optimisation of the resources available for cancer research, broadly mentions a limited list of preanalytic variables related to the donor or sample collection/processing. It has thereby been effective in raising the overall awareness and quality of research involving biospecimens.

Although this is an important start, many parameters and variables of interest, including choice of biospecimens and clinical data, are cancer-type-specific. Thus, universal biobanking standards are not necessarily applicable to every cancer type and should be adapted to each specific disease. The importance of cancer-specific harmonisation of biobanking standards is highlighted by the cancer genome atlas (Kandoth *et al*, 2013), which now contains over 532 EC samples with RNA sequencing, copy number variation, proteomic, mutation and microarray data. However, the extremely limited clinical data accompanying most of these samples and data sets severely affects the ability of researchers to draw clinically applicable information.

Therefore, EC-specific standardisation of the collection of biospecimens with distinctive and relevant accompanying clinical data sets is a fundamental unmet need in improving future EC research. This, we believe, will facilitate future large-scale internationally collaborative research into EC, which could lead to improved biomarker and target treatment discovery. Similar harmonisation projects have already been successfully implemented for other gynaecological conditions such as endometriosis – World Endometriosis Research Foundation Endometriosis Phenome and Biobanking Harmonisation Project and Ovarian Cancer Research Program (Wiegand *et al*, 2010; Heravi-Moussavi *et al*, 2012; Fassbender *et al*, 2014; Rahmioglu *et al*, 2014; Vitonis *et al*, 2014).

With this background, we initiated our study (Harmonisation of biobAnking STandards in Endometrial caNcer research – HASTEN) to achieve consensus among EC researchers; standardise the collection, processing and storage of all relevant biospecimens; and the accompanying clinical data for EC research through a joint effort with patients, surgeons/physicians/pathologists and the personnel of biobanks. We aimed to develop standards: standard operative procedure tools with a minimum and standard data set to be regularly updated and universally available for future researchers in EC.

## Materials and methods

**Figure 1:**
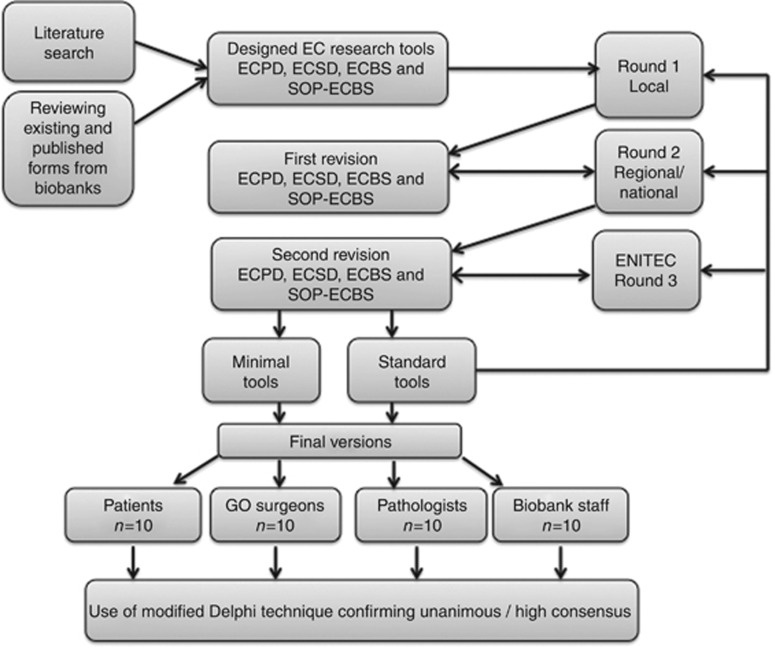
**Flow chart illustrating our workflow in designing the EC research tools and the method of generating consensus (Endometrial cancer (EC), European Network of Individualised Treatment in Endometrial Cancer (ENITEC), Endometrial Cancer Patient Data Collection Tool (ECPD), Endometrial Cancer Surgical Data Collection Tool (ECSD), Endometrial Cancer Biospecimen Tool (ECBS), standard operating procedure for collection, processing and storage of tissue and fluid for EC research (SOP-ECBS)).**

The method used to design the final tools in HASTEN is summarised in the flow diagram (Figure 1). We used a modified Delphi system to analyse and confirm the final consensus.

### Generation of the initial tools

#### Literature search

We performed a systematic review of the literature using the keywords ‘Endometrial Cancer’, ‘risk factors’, ‘age of presentation’, ‘parity’, ‘menopausal status’, ‘metformin’, ‘progestogens or Mirena’, ‘hormone replacement therapy’, ‘polycystic ovary syndrome’, ‘tamoxifen’, ‘bowel cancer’, ‘colorectal cancer’, ‘breast cancer’, ‘diabetes’, ‘hypertension’, ‘ethnicity’, ‘anthropometric assessment’, ‘smoking’, ‘standard operating procedure’ and ‘endometrium’, ‘blood or plasma or serum’, ‘saliva’, ‘urine’, ‘endometrial fluid’, ‘peritoneal fluid’, ‘biobank best practices’ ‘histopathology markers’, ‘outcomes’, ‘biomarkers’, ‘Laboratory processing procedures of tissue, blood and body fluids’, ‘biobanking standards’, ‘SOP’s for collection of tissue, fluids, blood, saliva, urine’ in Scopus, Discover and PubMed databases. The literature search was limited to studies published in the past 10 years. Out of 3464 papers identified in the initial search, 413 papers were selected for further detailed scrutiny based on the following inclusion criteria:

1. Papers that investigated how the aforementioned factors affect an individual’s risk of developing EC.
2. Publications that proposed standard operating procedures (SOPs) or best practices for the collection, storage and processing of the different tissues or fluids.
3. Papers in English language only.
4. Papers available as full text via all available resources to the authors (e.g., online resources or library facilities at Liverpool Women’s Hospital (LWH), University of Liverpool, British Medical Association or Royal College of Obstetricians and Gynaecologists.

We further conducted manual searches for the relevant manuscripts referenced in these selected papers and the relevant guidelines from the large biorepositories.

## Further development of the tools

### First local consultation

The local team at Liverpool, comprising of four members of surgical gynaecological oncology team, four Macmillan clinical cancer nurse specialists, two clinical academics with an interest in EC research, two pathologists, two biobank staff members and a medical student, developed the three forms (patient data collection tool, surgical data collection tool, biospecimen form) and a standard operative procedure. These forms and the SOP were based on: (a) the information gathered in the literature search; (b) by considering the forms that were already in use in LWH/University of Liverpool biobank to collect biospecimens and data in EC research studies. Liverpool Women’s Hospital is a tertiary referral regional cancer centre for gynaecological cancers, and is part of the Cheshire and Merseyside strategic clinical networks, which serves a population of 2.4 million. The age-standardised incidence rate of EC in the Merseyside and Cheshire cancer network is 18.3 per 100 000 female members of the population (NCIN, 2013; Gynae Clinical Network Constitution, 2014–2015). (c) Standard operating procedures developed by the National Institutes of Health, Human Endometrial Tissue and DNA Bank for the collection, transport and storage of human endometrial tissue and blood samples of women undergoing endometrial biopsy or hysterectomy for non-malignant indications (Sheldon *et al*, 2011). (d) Sample handling and storage protocol published by the UK biobank to collect urine and blood samples (Elliott *et al*, 2008). UK biobank is a major national and international health resource, which was established by Wellcome trust, Medical Research Council, Department of Health, Scottish Government and The Northwest Regional Development Agency. The main aim of this was to improve prevention, diagnosis and treatment of many illnesses such as cancer, heart disease, stroke, diabetes, arthritis, osteoporosis and dementia.

The forms were revised and amended based on local consultation.

### Second regional/national consultation

The modified versions of the three forms and the SOP mentioned above were disseminated among three regional and eight national research centres involved in EC research in the United Kingdom and forms were revised integrating their feedback and as a result, two different tools, a minimal and a standard tool were developed. This pragmatic and inclusive approach provides guidance for collecting either a minimal or the ideal ‘standard’ data set considering the available resources.

### Third European consultation

The modified forms were then circulated to all researchers adhering to the European Network of Individualised Treatment in Endometrial Cancer (ENITEC) and were further revised according to feedback received. The revised tools were presented at the annual ENITEC face-to-face meeting in June 2016, where the minimal form was unanimously approved by all 47 attendees. Some further modifications were suggested for the standard tool, which was revised accordingly and the revised forms were repopulated to all participated in the consultations rounds 1–3 to obtain their final approval.

### Consensus generation

A modified Delphi system was used to generate consensus regarding the final adapted tools. For this, the forms were disseminated to a group of selected panel members of representing all stakeholders included in all previous rounds, including patients, gynaecological oncologists, researchers, pathologists and biobank staff, randomly selected from the participants of the consultation (*n*=40) to evaluate and score the tools using a scoring sheet recording their agreement.

### Statistical analysis

The consensus was quantified using a modified Delphi technique and we have reported the median with an interquartile range and also percentages for each category of the Likert scale. A nine-point Likert scale was used, except for the patient data tool where the scale was reduced to five points to reduce complexity for patients.

## Results

### Final tools

#### ECPD collection tool

**Figure 2:**
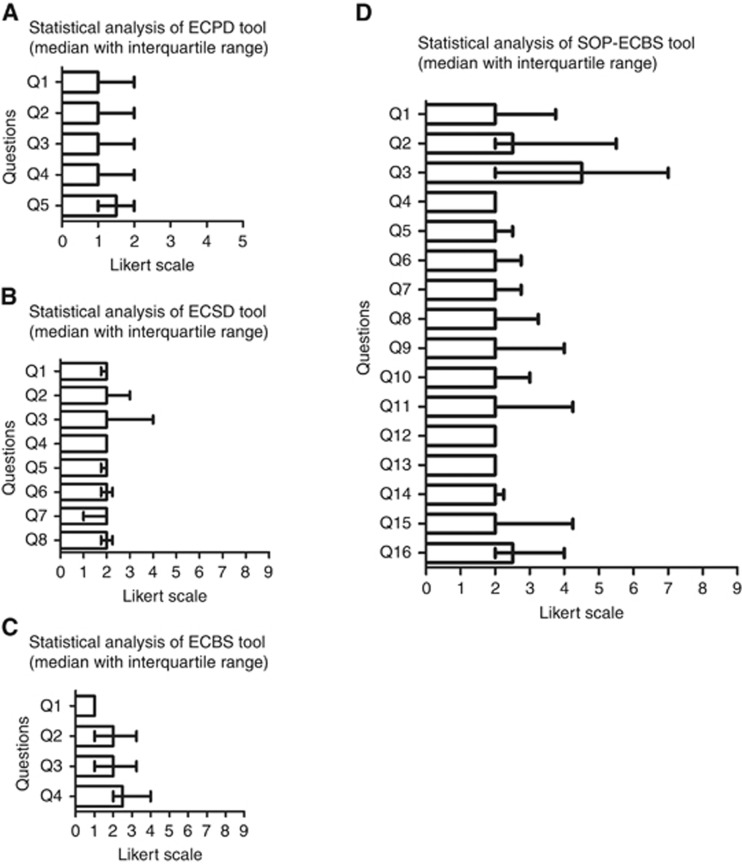
**Statistical analysis of Endometrial Cancer Patient Data Collection Tool (ECPD), Endometrial Cancer Surgical Data Collection Tool (ECSD), Endometrial Cancer Biospecimen Tool (ECBS) and standard operating procedure for collection, processing and storage of tissue and fluid for EC research (SOP-ECBS) tools.**

A patient-friendly data collection tool (EC patient data (ECPD)) was devised to capture many important demographic variables that are directly relevant to EC research that can only be accurately recalled by the patient herself. For example, the available literature suggests that >20 kg of adult weight gain to be independently associated with increased risk of EC (Friedenreich *et al*, 2007) and this information is unlikely to be obtained easily other than directly from the patient. Many other risk factors for EC such as the age of presentation, the postmenopausal status, polycystic ovarian disease (Fearnley *et al*, 2010), nulliparity (Schonfeld *et al*, 2013), early age of menarche (Gong *et al*, 2015), family history of hereditary lynch syndrome-related cancers (Boilesen *et al*, 2008), past history of lynch syndrome-related cancers, medical conditions such diabetes (Friberg *et al*, 2007), previous use of tamoxifen (Bergman *et al*, 2000), hormone replacement therapy use (Beral *et al*, 2005) and exercise habits have been included in the tool. Some other factors with inconclusive links to EC at present such as smoking (Lindemann *et al*, 2008) were also included in anticipation of their confirmation in appropriate future studies. Table 1 and Figure 2 illustrate the outcome of the final round of consensus. Score for each question in ECPD was obtained using the Likert scale, which assesses the acceptability and usability (*n*=10). Among the panel members, only 2% were undecided on the clarity of the questions in social history section, and overall, 98% patients agreed that the tool was easy to use (Supplementary Figure S1).

#### ECSD collection tool

The EC surgical data (ECSD) tool included salient demographic, histological and pre/postoperative features. Demographic features such as body mass index (BMI) were included. Body mass index instead of waist-to-hip ratio or waist circumference was chosen because of its universal use and reproducibility. Although all anthropometric assessments (BMI, waist-to-hip ratio, waist and hip circumferences) are found to be strongly associated with increased risk of EC (Friedenreich *et al*, 2007), accurate data on waist-to-hip ratio or waist circumferences require additional effort using the same reference points by health-care team and thus accurate data collection is unlikely to be universally feasible. In a recent study (Painter *et al*, 2016), BMI was found to be a causal factor and was associated with EC compared with waist-to-hip ratio. The preoperative imaging details are helpful to assess the spread locally and to rule out distant metastases. Discordance between endometrial biopsy and final histology results has been shown to be associated with poorer survival outcome (Werner *et al*, 2013); hence, preoperative biopsy results are important. Staging details including operative findings and final histopathologic details after surgery are important when correlating with outcomes. Immunohistochemical biomarkers can be used to distinguish ECs from ovarian or cervical or other malignancies, but importantly also as prognostic biomarkers that are associated with clinical outcome (Li *et al*, 2013; Kamal *et al*, 2016). Information when collected in a standard way together with biosamples will naturally increase the internal and external validity of the generated data. The patient data collection, including follow-up and accurate documentation of cause of demise, should be updated regularly until the completion of standard follow-up period (either 3 years (minimum) or 5 years, depending on local practice). The form is arranged into three sections:

1. Surgical data: Completed at the time of sample collection.
2. Histopathology details: Completed after final staging and treatment.
3. Outcome data: Documented during follow-up and finally at the end of follow-up

The results of final consensus are as shown in Table 2 and Figure 2, wherein we have calculated the median with an interquartile range. There was a high level of agreement among the panellists for all sections, except that a number of the respondents considered sections on the history, antecedent biopsy details and sample collection details to be not relevant. Overall, 96.25% of panel members agreed on different aspects of the tool (Supplementary Figure S1).

#### EC Biospecimen tool

Variations in the collection methods and biobanking conditions (processing and storage) may alter the molecular composition, expression and stability of biomarker profile (Zander *et al*, 2014); thus, consistency and strict adherence to standard operating procedures is vital (Moore *et al*, 2011). Therefore, biobank staff with applied experience and knowledge on clinical biobanking participated in designing, revising and obtaining final consensus on the biospecimen form. Only few respondents felt that the tissue processing (both uterine and extrauterine) section of the form was difficult to understand, while all respondents agreed on the relevance and clarity of all other sections. Overall, there was a 94% level of agreement on the different aspects of this tool. The detailed results were as shown in Table 3, Figure 2 and Supplementary Figure S1.

### Standard operating procedure for collection, processing and storage of tissue and fluid for EC research

Different tissue types (both uterine and extrauterine) and body fluid types are studied in EC research. The routine investigations of these biospecimens may involve extraction of protein, RNA and DNA to be evaluated using a variety of techniques such as proteomics, genomics and metabolomics. The final SOP was designed amalgamating a number of available separate, detailed methodological protocols (e.g., for centrifugation, filtration, addition of preservatives, as well as storage temperatures). Availability of such information from a biobank will allow the scientists to accurately interpret their data, for example, to examine the metabolic profile of samples such as blood, tissue, endometrial fluid or aspirate and detect disease-specific changes with confidence, especially in multicentre studies (Assfalg *et al*, 2008; Bernini *et al*, 2009). Studies examining hormones are of major relevance to the endometrium, and in addition to more traditional samples such as blood, some have studied noninvasive specimens including saliva and urine (Shirtcliff *et al*, 2001). Noninvasive tests are of a particular interest in clinical research and future work is expected to focus more on them.

The outcome details of the final round of consensus regarding the standard operating procedure for collection, processing and storage of tissue and fluid for EC research (SOP-ECBS) are as presented in Table 4 and Figure 2. There was a general agreement on the user-friendliness and relevance of the tool. Few panellists responded that tissue and blood collection details could be modified further for clarity. Overall, 83.75% of panellists agreed, 8.75% were undecided and 7.5% disagreed with different sections of this tool.

## Discussion

We have developed evidence-based standard data collection forms ECPD, ECSD (minimal), ECSD (Standard) and an SOP-ECBS with inclusive participation and approval of all stakeholders in EC cancer biobanking. The final tools were approved by a large multidisciplinary team of reviewers and after reaching consensus (see Supplementary Figure S1), they are published as Supplementary Information with this open access manuscript. They will therefore be freely available to all EC researchers internationally. These tools provide a means by which to reduce confounding factors in the collected data and facilitate larger multicentre studies.

Our choice of the exact information to collect was based on critical appraisal of the best available evidence. Where no published evidence was available, consultation of the experts’ opinion and the SOPs of the larger biobanks were considered. The centrifugation speed in processing blood was one such example.

We have used a modified Delphi technique, with multiple alterations from the standard technique, including multiple rounds of feedback, which allows the same panel members to reassess or reconsider initial judgment, participant anonymity, controlled feedback and statistical analysis to interpret data between the rounds. Similar variations to original Delphi system, for example, restricting the ability of the experts to respond to the original question and alterations in the expert groups, as well as changing the end point, have been used previously (Thompson, 2009).

Repeated use of a homogenous panel was unjustifiable for our research aims for the following reasons. Our endeavour was to generate separate forms for diverse end points, for example, patient data collection, surgical data collection, tissue processing information and the standard operative procedures. These obviously required panel members of diverse backgrounds, with different fields of expertise and therefore our panellists were not a homogenous group.

The main deviation from the classic technique was the number of consultation rounds and the end point. Our first two rounds were descriptive to generate opinions and ideas from different expert panels. We included their feedback to generate the finalised forms and SOP. In the final round of the consensus, we distributed a score sheet to each of the panellists along with the forms to evaluate their agreement with the final tools. Our final panel included stakeholders representing those involved in all previous panels. The high percentage of agreement observed with the statistical analysis of data obtained from the third and final round precluded the need for any further consensus rounds.

As more detailed, standardised surgical data collection will allow a comprehensive assessment of the relationship between the surgical phenotypical data with the outcomes of treatments, we strongly advise the use of the standard rather than the minimal ECSD tool. However, if the collection or quality of the large set of data or specimens cannot be guaranteed, the minimal set should be employed. We plan to regularly update these tools in the future through information obtained by feedback and review of future literature, initially on a yearly basis and 5 yearly thereafter. Future considerations in the context of our initiatives include creating an internationally funded web-based central database system allowing voluntary deposition of the information on all biospecimens collected by EC researchers worldwide, which will be easily accessible to all. This approach, we believe, will reduce costs and time spent by individual units while increasing the credibility of the data generated and will offer a transparent, common platform for newer collaborations.

‘Molecular Pathological Epidemiology’ (MPE) integrates pathology and epidemiology to understand the interrelationships between exogenous and endogenous factors that affect carcinogenesis, progression and response to treatment. It is a constantly evolving field in cancer research (Ogino and Stampfer, 2010). Statistical methods have also been developed to consider both molecular pathology and epidemiology to ensure novel discoveries with high clinical impact. However, the generation of such high-impact MPE studies are impeded by similar challenges including selection and recall bias, measurement errors and misclassification comparable to the traditional molecular biological studies (Hughes *et al*, 2012; Campbell *et al*, 2017). Variability in tissue retrieval rate and sample sizes leads to random and non-random selection bias, resulting in large variation of an effect estimate with wide confidence intervals and publication bias (Ogino *et al*, 2011, 2016). The use of our tools by EC biobanks will provide means with which to streamline the collection of a large amount of standardised quality assured material from well-phenotyped patients. This will in turn facilitate adequately powered studies, giving high clinical impact while also facilitating high-quality research that is attainable within an acceptable timescale.

## Figures and Tables

**Table 1:** Outcome of the final round of consensus for ECPD Tool using 5-point Likert scale

		**Percentages of responses (%)**				
**Statements in the score sheet for patients**	**Score, median (IQR)**	**Strongly agree**	**Agree**	**Undecided**	**Disagree**	**Strongly disagree**
The information asked in personal history is easy to fill	1 (1–2)	60	40	0	0	0
The questions in medical history section are easy to understand and fill	1 (1–2)	60	40	0	0	0
The questions in past history are easy to understand and fill	1 (1–2)	60	40	0	0	0
The questions in social history section are easy to understand and fill	1 (1–2)	60	30	10	0	0
Overall, the form is easy to understand and does not take much time to fill it	1.5 (1–2)	50	50	0	0	0

Abbreviations: ECPD=Endometrial Cancer Patient Data; IQR=interquartile range.

IQR, five-point Likert scale: Strongly agree, Agree, Undecided, Disagree and Strongly disagree; *n*=10.

**Table 2:** Outcome of the final round of consensus for ECSD Tool using nine-point Likert scale

		**Percentages of responses (%)**								
**Questions in the score sheet for gynaeoncologists**	**Score, median (IQR)**	**Strongly agree**	**Agree**	**Moderately agree**	**Mildly agree**	**Undecided**	**Mildly disagree**	**Moderately disagree**	**Disagree**	**Strongly disagree**
Is the general information about patient relevant?	2 (1.75–2)	20	80	0	0	0	0	0	0	0
Is the section on history relevant?	2 (2–3)	10	60	20	0	0	0	0	10	0
Are the Imaging details relevant and sufficient?	2 (2–4)	10	80	0	30	0	0	0	0	0
Are Antecedent biopsy details relevant?	2 (2–2)	10	80	0	0	0	0	0	0	10
Is the Operative findings section relevant?	2 (1.75–2)	20	80	0	0	0	0	0	0	0
Is the Histopathology type details section relevant and sufficient?	2 (1.75–2.25)	20	60	10	10	0	0	0	0	0
Is the Sample collection details section easy to complete?	2 (1–2)	40	50	0	0	0	0	0	0	10
Are Outcome details relevant?	2 (1.75–2.25)	20	60	10	10	0	0	0	0	0

Abbreviations: ECSD=Endometrial Cancer Surgical Data; IQR=interquartile range.

IQR, nine-point Likert scale: Strongly agree, Agree, Moderately agree, Mildly agree, Undecided, Mildly disagree, Disagree and Strongly disagree; *n*=10.

**Table 3:** Outcome of the final round of consensus for ECBS Tool using nine-point Likert scale

		**Percentages of responses (%)**								
**Questions in the score sheet for biobank staff**	**Score Median (IQR)**	**Strongly agree**	**Agree**	**Moderately agree**	**Mildly agree**	**Undecided**	**Mildly disagree**	**Moderately disagree**	**Disagree**	**Strongly disagree**
Sample ID–Is this relevant?	1 (1–1)	90	10	0	0	0	0	0	0	0
Methods of tissue processing (Endometrium)–Is this section easy to understand?	2 (1–3.25)	40	30	10	10	0	0	0	10	0
Methods of tissue processing (Extra uterine tissue)–Is this section easy to understand?	2 (1–3.25)	40	30	10	10	0	0	0	10	0
Methods of fluid processing (Endometrial/Peritoneal/Blood/Saliva/Urine)–Is this section easy to understand?	2.5 (2–4)	10	40	10	40	0	0	0	0	0

Abbreviations: ECBS=Endometrial Cancer Biospecimen; IQR=interquartile range.

IQR, nine-point Likert scale: Strongly agree, Agree, Moderately agree, Mildly agree, Undecided, Mildly disagree, Disagree and Strongly disagree; *n*=10

**Table 4:** Outcome of the final round of consensus for SOP-ECBS Tool using nine-point Likert scale

		**Percentages of responses (%)**								
**Questions in the score sheet for pathologists**	**Score Median (IQR)**	**Strongly agree**	**Agree**	**Moderately agree**	**Mildly agree**	**Undecided**	**Mildly disagree**	**Moderately disagree**	**Disagree**	**Strongly disagree**
Is section: Processing and storage materials, relevant and easy to understand?	2 (2–3.75)	10	50	20	0	0	20	0	0	0
Is section: Collection–Tissue, relevant and easy to understand?	2.5 (2–5.5)	10	40	10	10	10	0	20	0	0
Is section: Collection–Blood, relevant and easy to understand?	4.5 (2–7)	10	30	0	10	10	10	30	0	0
Is section: Collection – Urine, relevant and easy to understand?	2 (2–2)	10	80	10	0	0	0	0	0	0
Is section: Collection – Saliva, relevant and easy to understand?	2 (2–2.5)	10	70	0	10	10	0	0	0	0
Is section: Collection – Peritoneal fluid, relevant and easy to understand?	2 (2–2.75)	0	80	0	0	20	0	0	0	0
Is section: Collection – Endometrial fluid/uterine aspirates, relevant and easy to understand?	2 (2–2.75)	0	80	0	0	20	0	0	0	0
Is section: Sample processing – Tissue, relevant and easy to understand?	2 (2–3.25)	0	70	10	10	10	0	0	0	0
Is section: Sample processing – Blood, relevant and easy to understand?	2 (2–4)	0	60	10	20	10	0	0	0	0
Is section: Sample processing – Urine, relevant and easy to understand?	2 (2–3)	0	70	20	0	0	10	0	0	0
Is section: Sample processing – Saliva, relevant and easy to understand?	2 (2–4.25)	0	60	10	10	10	10	0	0	0
Is section: Sample processing – Peritoneal fluid, relevant and easy to understand?	2 (2–2)	10	80	0	0	10	0	0	0	0
Is section: Sample processing – Endometrial fluid/uterine aspirates, relevant and easy to understand?	2 (2–2)	10	80	0	0	10	0	0	0	0
Is section: Storage and data recording, relevant and easy to understand?	2 (2–2.25)	10	70	10	0	0	10	0	0	0
Is section: Freezer check, relevant and easy to understand?	2 (2–4.25)	0	60	10	10	10	10	0	0	0
Is section: Checklist, relevant and easy to understand?	2.5 (2–4)	0	50	10	30	10	0	0	0	0

Abbreviations: IQR=interquartile range; SOP-ECBS=standard operating procedure for collection, processing and storage of tissue and fluid for EC research.

IQR, nine-point Likert scale: Strongly agree, Agree, Moderately agree, Mildly agree, Undecided, Mildly disagree, Disagree and Strongly disagree; *n*=10.
