# Supplementary material for: Harmonisation of biobanking standards in endometrial cancer research
Source: Br J Cancer. 2017 Jun 29;117(4):485–93. doi: 10.1038/bjc.2017.194 (PMC5558683; doi:10.1038/bjc.2017.194)
Supplement: Supplementary Document 1 [file bjc2017194x1.docx]

Endometrial Cancer Patient Data (ECPD) Collection Tool:

The doctors and nurses at the Hospital are collecting information from patients who are undergoing tests and treatment for endometrial cancer, along with samples for cancer research. We hope to use this information to improve treatments for patients in the future. We would be very grateful if you could complete this short questionnaire before you have your operation. The questionnaire will take 10 minutes to complete. If you require help to complete the questionnaire please ask a relative, friend or a nurse or doctor.

Thank you for your cooperation.

Age - ______ Ethnicity - ______________________

Weight - _____ Weight at 18 yrs of age (approximate) - ______Height- ________

Have your periods stopped? Yes/ No

Age periods stopped (menopause)? ____

What age did your periods start?________

Were /are your periods- regular / irregular

How many days were/are in between your periods? ____

How many days do/did you bleed for? ____

Date of last menstrual period -

How many pregnancies have you had? ____

How many children do you have? ____

How old were you when you had your first pregnancy? _____

Did you have any problems becoming pregnant? Yes / No

Have you ever had IVF? Yes / No

Have you ever had any serious infectious disease? - Nil / HIV / Hepatitis B / Hepatitis C / Syphilis

Have you ever had pelvic inflammatory disease? - Yes / No

Is there a family history of cancer in any of your first degree relatives (eg. Mother/father/sister)? - None / Bowel / Breast / Ovarian / Thyroid / Other _______________________________________

Please turn over

Have you had cancer in the past? – Yes / No

If Yes - Bowel / Breast / Ovarian / Thyroid / Other_________________

Please circle any of these medical conditions that you have and inform us of any other medical conditions you have been diagnose with – Type I Diabetes / Type 2 Diabetes / Polycystic ovarian syndrome / Thyroid disorders / High Blood Pressure/ High cholesterol ___________________________________________________________________

___________________________________________________________________

Please circle any of these medications that you have ever been on and inform us of any other medications you are currently on – Oral Progesterone/ Mirena/Contraceptive pill / Tamoxifen / Metformin / HRT / Other ___________________________________________________________________

___________________________________________________________________

Do you smoke? Yes / No / Ex smoker / E-cigarette

If you have ever smoked how many did/do you smoke a day?_______ For how many yrs?_______

Do you drink any alcohol? – Yes / No/ In the past

If you have ever drunk alcohol, type of drink and approximately how much would you drink per week? ____________ (e.g. 2 glasses of wine per week or 1 bottle of beer per week)

How often do you exercise (any form for 30 mins or above)? Daily / Once or twice a week / Once a month / Less than once a month / stopped / Never

If stopped, the reason for stopping: ____________________________________________________

What investigations you have prior to diagnosis of endometrial cancer? Ultrasound / Hysteroscopy and biopsy (camera and biopsy) / Biopsy only / CT scan / MRI scan /Not known

Many Thanks for providing the above valuable and confidential information, this will be stored in our biobank database and will be used for cancer research purposes only.
