# Supplementary material for: Harmonisation of biobanking standards in endometrial cancer research
Source: Br J Cancer. 2017 Jun 29;117(4):485–93. doi: 10.1038/bjc.2017.194 (PMC5558683; doi:10.1038/bjc.2017.194)
Supplement: Supplementary Document 2 [file bjc2017194x2.docx]

Endometrial Cancer Biospecimen (ECBS) tool: **To be filled by lab staff**

Sample ID:____________________________

**Processing and Storage details**

**Samples received: Date __/__/__ Time __:__hrs**

| **Tissue -**  **Endometrium /**  **Extraendometrial** |  | **Blood** |  | **Endometrial**  **fluid** |  | **Peritoneal /ascitic**  **fluid** |  | **Urine** |  | **Saliva** |  |
| --- | --- | --- | --- | --- | --- | --- | --- | --- | --- | --- | --- |
| **NBF** |  | **Whole** |  | **Plain** |  | **Plain** |  | **Plain** |  | **Plain** |  |
| **Culture media** |  | **Serum** |  | **RNA stabilisation additives** |  | **RNA stabilisation additives** |  | **RNA stabilisation additives** |  | **RNA stabilisation additives** |  |
| **RNA Later** |  |  |  |  |  |  |  |  |  |  |  |
| **Frozen tissue** |  |  |  |  |  |  |  |  |  |  |  |
| **Other (state)** |  |  |  |  |  |  |  |  |  |  |  |

**Details of tissue processing:**

**Endometrium**

| **Tissue**  **Processing** | **Number of blocks/pieces of tissue** | **Processing start time & Date** | **Date & time**  **in storage** | **Storage**  **Location** |
| --- | --- | --- | --- | --- |
| **Frozen Tissue** |  |  |  |  |
| **RNA later** |  |  |  |  |
| **Paraffin embedded** |  |  |  |  |

**Extra uterine tissue**

| **Tissue Processing** | **Number of blocks/pieces of tissue** | **Processing start time & Date** | **Date & time**  **in storage** | **Storage**  **Location** |
| --- | --- | --- | --- | --- |
| **Frozen Tissue** |  |  |  |  |
| **RNA later** |  |  |  |  |
| **Paraffin embedded** |  |  |  |  |

**Please turn over**

**Details of fluid processing:**

**Endometrial fluid**

| **Number of Aliquots** | | | **Total Volume** | **Processing start time** | **Date & time in storage** | **Storage location** |
| --- | --- | --- | --- | --- | --- | --- |
| **Amount** | | |  |  |  |  |
| **50 µl** | **250 µl** | **1 ml** |  |  |  |  |
|  |  |  |  |  |  |  |
|  |  |  |  |  |  |  |
|  |  |  |  |  |  |  |

**Peritoneal / Ascitic fluid**

| **Number of Aliquots** | | | **Total Volume** | **Processing start time** | **Date & time in storage** | **Storage location** |
| --- | --- | --- | --- | --- | --- | --- |
| **Amount** | | |  |  |  |  |
| **50 µl** | **250 µl** | **1 ml** |  |  |  |  |
|  |  |  |  |  |  |  |
|  |  |  |  |  |  |  |
|  |  |  |  |  |  |  |

**Blood samples**

| **Number of Aliquots** | | | | **Total Volume** | **Processing start time** | **Date & time in storage** | **Storage location** |
| --- | --- | --- | --- | --- | --- | --- | --- |
| **Amount** | **50 µl** | **250 µl** | **1 ml** |  |  |  |  |
| serum |  |  |  |  |  |  |  |
| whole |  |  |  |  |  |  |  |
| plasma |  |  |  |  |  |  |  |

**Saliva samples**

| **Number of Aliquots** | | | **Total Volume** | **Processing start time** | **Date & time in storage** | **Storage location** |
| --- | --- | --- | --- | --- | --- | --- |
| **Amount** | | |  |  |  |  |
| **50 µl** | **250 µl** | **1 ml** |  |  |  |  |
|  |  |  |  |  |  |  |
|  |  |  |  |  |  |  |
|  |  |  |  |  |  |  |

**Urine samples**

| **Number of Aliquots** | | | **Total Volume** | **Processing start time** | **Date & time in storage** | **Storage location** |
| --- | --- | --- | --- | --- | --- | --- |
| **Amount** | | |  |  |  |  |
| **50 µl** | **250 µl** | **1 ml** |  |  |  |  |
|  |  |  |  |  |  |  |
|  |  |  |  |  |  |  |
|  |  |  |  |  |  |  |

Any variations or deviations from the SOP, problems, or issues:

**________________________________________________________________________________________________________________________________________________________________________________________________________________________________________________________**
