# Supplementary material for: Harmonisation of biobanking standards in endometrial cancer research
Source: Br J Cancer. 2017 Jun 29;117(4):485–93. doi: 10.1038/bjc.2017.194 (PMC5558683; doi:10.1038/bjc.2017.194)
Supplement: Supplementary Document 3 [file bjc2017194x3.docx]

### **Endometrial Cancer Surgical Data (ECSD) Collection Tool – Minimal**

### Study ID no:

Age _____________

Parity_______

BMI ____________

**Presenting symptom** – PMB / None

**Antecedental endometrial biopsy if definitive treatment:**

Yes 🞎 No 🞎

If Yes – date of biopsy ___/__/__

**Operation –**

TLH/LAVH/TAH/VH

Diagnostic / Curative / Palliative

Findings: Uterus Normal ⎕ Abnormal ⎕

Tubes Normal ⎕ Abnormal ⎕

Ovaries Normal ⎕ Cysts⎕ Abnormal⎕

Extrauterine extension –

Cervical involvement⎕

Adnexae / Parametrium⎕

Lymph Nodes⎕

Locoregional metastasis (rectum/bladder) ⎕

Bowel⎕Liver⎕Omentum⎕

**Histopathologic type** –

Endometrioid / serous / clear cell / mucinous / carcinosarcoma/undifferentiated/Mixed /Other_____________

Grade – 1 / 2 / 3

LVSI present⎕ LVSI absent ⎕

FIGO stage – I / II / III / IV

Nodes sampled: No/ Yes, Pelvic ⎕ Number of nodes - ___, Positive___ / Negative___

Para aortic ⎕ Number of nodes - ___, Positive___ / Negative___

Biomarkers performed: Yes ⎕ / No ⎕

P53 ⎕ PTEN ⎕ ER ⎕ PR ⎕ HER 2 ⎕ P16 ⎕ MLH1 ⎕ MSH2 ⎕ MSH6 ⎕ PMS2 ⎕ KRAS⎕ PIK3Ca ⎕ HE4 ⎕ Stathmin ⎕ L1CAM ⎕

Status/ score:___________

**Sample collection –**

Timing of sample collection – At time of diagnosis ⎕

Primary surgery⎕

Relapse⎕

Samples in:

NBF ⎕ PBS⎕ RNA later⎕ Snap frozen⎕

Sample type

⎕ Uterine - Pipelle ⎕ Currettings⎕ surgical resection ⎕

Dt __/__/__ Time __:__

⎕ Extra uterine Dt __/__/__ Time __:__

⎕ Urine Dt __/__/__ Time __:__

⎕ Blood Dt __/__/__ Time __:__

⎕ Endometrial fluid Dt __/__/__ Time __:__

⎕ Peritoneal or ascitic fluid Dt __/__/__ Time __:_

**Primary treatment –** Surgery⎕ Radiotherapy⎕ Chemotherapy ⎕

**Outcome for patient:** Date of last cancer follow up__/__/__

Date of Death __/__/__

Recurrence- Local / distant /unknown, Date __/__/__
