# Supplementary material for: Harmonisation of biobanking standards in endometrial cancer research
Source: Br J Cancer. 2017 Jun 29;117(4):485–93. doi: 10.1038/bjc.2017.194 (PMC5558683; doi:10.1038/bjc.2017.194)
Supplement: Supplementary Document 4 [file bjc2017194x4.docx]

### **Endometrial Cancer Surgical Data (ECSD) Collection Tool - Standard:**

Age _____________ Parity_______

Performance status – 0 / 1 / 2 / 3

BMI ____________

**Presenting symptom** – PMB / Other

**Imaging** –

US /MRI / PET CT

Findings: Tumour size ______ cm(MRI or USS)

Myometrial invasion <50%⎕ >50% ⎕

Extrauterine extension - Cervical involvement⎕

Adnexae⎕

Lymph Nodes⎕

Locoregional metastasis (rectum/bladder) ⎕

Distant metastasis⎕

Other⎕

**Antecedent endometrial biopsy if definitive treatment:**

Yes 🞎 No 🞎 date of biopsy ___/__/__

**Operation –**

**TLH / LAVH / TAH /VH/Hysteroscopy**

Anaesthetic time __:__ hrs

Diagnostic / Curative / Palliative

Findings Uterus - Normal ⎕ Abnormal ⎕

Tubes - Normal ⎕ Abnormal ⎕

Ovaries - Normal ⎕ Cysts⎕ Abnormal⎕

Extrauterine extension - Cervical involvement⎕

Adnexae / Parametrium⎕

Lymph Nodes⎕

Locoregional metastasis (rectum/bladder) ⎕

Bowel⎕Liver⎕Omentum⎕

**Histopathologic type** –

Endometrioid / serous / clear cell / mucinous / carcinosarcoma/undifferentiated/Mixed /Other_____________

Grade – 1 / 2 / 3

Lymphovascular invasion Present⎕ Absent⎕

FIGO stage – I / II / III / IV

Nodes sampled: No/ Yes, Pelvic⎕ Number of nodes - _____ , Positive___ / Negative ___

Para aortic⎕ Number of nodes - _____ , Positive___ / Negative ___

Biomarkers performed: Yes ⎕ / No ⎕

P53 ⎕ PTEN ⎕ ER ⎕ PR ⎕ HER 2 ⎕ P16 ⎕ MLH1 ⎕ MSH2 ⎕ MSH6 ⎕ PMS2 ⎕ KRAS⎕ PIK3Ca ⎕ HE4 ⎕ Stathmin ⎕ L1CAM ⎕

Status/ score:____________

**Sample collection –**

Timing of sample collection – At time of diagnosis ⎕

Primary surgery⎕

Relapse⎕

Samples in:

NBF ⎕ PBS⎕ RNA later⎕ Snap frozen ⎕

Sample type collected

A – Uterine - Date __/__/__ Time __:__

-Tissue / aspirate

-Hysteroscopic/ laparoscopic / open

-Medium used - saline /gas /glycine

-Location of sample collection - outpatient / general anaesthetic

-Pipelle/ curettage/ after hysterectomy

-Collected - Prior to uterine manipulator/ pre hysteroscopy /

Post hysteroscopy/ post hysterectomy

B – Extra uterine - Date __/__/__ Time __:__

-Tissue from metastatic lesion – source______________

-Laparoscopic / open

-Collected - Prior to uterine manipulator/ pre hysteroscopy /

Post hysteroscopy/ post hysterectomy

C – Urine –Collected – Date __/__/__ Time __:__

Collected pre theatre / intraoperative / post theatre

Mid stream sample / catheter sample

D - Blood–Collected – Date __/__/__ Time __:__

Collected pre theatre / intraoperative / post theatre

E – Peritoneal fluid – Date __/__/__ Time __:__

Collected at laparoscopy / open procedure

F – Ascitic fluid – Date __/__/__ Time __:__

Collected at paracentesis / laparoscopy / open procedure

**Primary treatment –** Surgery⎕ Radiotherapy⎕ Chemotherapy ⎕

**Outcome for patient:** Date of last cancer follow up__/__/__

Date of Death __/__/__

Recurrence- Local / distant /unknown, Date __/__/__
