# Supplementary material for: Harmonisation of biobanking standards in endometrial cancer research
Source: Br J Cancer. 2017 Jun 29;117(4):485–93. doi: 10.1038/bjc.2017.194 (PMC5558683; doi:10.1038/bjc.2017.194)
Supplement: Supplementary Document 5 [file bjc2017194x5.docx]

**Standard Operating Procedures for collection, processing and storage of the tissue, blood, urine, saliva and fluid samples for endometrial cancer research (SOP-ECBS):**

**Processing and storage materials**:

Biospecimen form

Log sheet to record the sample data

Labels suitable for long term storage or Bar codes

**For frozen tissues:**

Cryosafe aliquot vials

RNA stabilising solution

Freezers -80^o^C

Liquid nitrogen (LN_2_) for snap freeze

Crushed ice (for transport prior to processing)

**For fixed tissues:**

Neutral buffered formalin 10 % (NBF), universal molecular fixative, paraffin wax, cassettes

**For blood:**

Blood collection tubes with EDTA and heparin for plasma, SST or no SST for serum

Crushed ice

Racks to hold the tubes in upright position

Temperature controlled centrifuge

Pipettes

Cryogenic vials with screw top gasket closure

Freezers -80°C or liquid nitrogen (LN_2_)

**For urine:**

Sterile urine collection container with leak proof cap

Crushed ice

Pipettes

Dipstick

Temperature controlled centrifuge

Aliquot vials with screw top gasket closure

Freezers -80^o^C or liquid nitrogen (LN_2_)

**For saliva:**

Sterile saliva container or manufacturer’s provided container for DNA

Crushed ice

Pipettes

Qiagen’s RNA Protect saliva kit

Temperature controlled centrifuge

Aliquot vials with screw top gasket closure

Freezers -80^o^C or liquid nitrogen (LN_2_)

**For peritoneal fluid:**

20 ml suction device or laparoscopic needle and a 20 ml syringe

Normal saline solution

Crushed ice

Transfer pipette

Volume adjustable pipette

Temperature controlled centrifuge

Aliquot vials with screw top gasket closure

Freezers – 80^o^C or colder / liquid nitrogen (LN_2_)

**For endometrial fluid or aspirates:**

Embryo transfer catheter and a 20 ml syringe or Cornier pipelle

Normal saline solution

Crushed ice, liquid nitrogen (LN_2_) or dry ice

Transfer pipette

Volume adjustable pipette

1.5 mL Eppendorf tubes

Temperature controlled centrifuge

Freezers - 80^o^C / liquid nitrogen (LN_2_)

**1 Collection**

- - Prepare all the necessary materials needed for storing and for recording data
  - Prepare all vials / tubes for surgical collection. Pre label each collection vial with unique identifier, participant ID, Date, time of collection and type of sample.
  - Record on the log sheet, date and time of collection.

**A Tissue:**

- 1. If samples are to be snap frozen immediately in liquid nitrogen (in a specific safe area near theatre) or once obtained, tissue samples should be placed in suitable pre-chilled containers on wet ice so they can be transported to the lab for processing (This will minimise autolysis of RNA and any *ex vivo* changes in RNA/protein expression profiles). Alternatively an RNA stabilisation solution can be used directly but this will be at the expense of being able to visualise tissue morphology by frozen sectioning. For hysterectomy specimens, the uterus should be transferred from theatre to the pathology department as quickly as possible to be opened and sampled by a pathologist. Again the sampled tissue should be placed into pre-chilled tubes and stored on wet ice prior to processing.
  2. Record type of tissue (uterine/ metastatic/ peritoneal/ myometrium/recurrent lesions (specify location)) collected: method used (curettage, endometrial sampling device, hysterectomy, hysteroscopy specimen, using cold scalpel or diathermy or harmonic device).
  3. Record whether endometrium collected 1) prior to anaesthesia, 2) after sedation but before anaesthesia, 3) after anaesthesia.
  4. Deposition of sample: Insert tissues in prepared, labelled vials / tubes and snap freeze as soon as possible in liquid nitrogen in / near theatre in their final vials. If delays >15 min are expected (especially if hysterectomy specimens), immerse tiny fragments in an RNA stabiliser solution if required. If sample needed for frozen section rather than RNA then freeze in liquid nitrogen cooled isopentane.

**B Blood**

- 1. Collect blood using standard withdrawal procedures and in adequate setting by a suitably qualified personnel.
  2. Collect samples after fasting for at least 10 hours and record the fasting time on the log sheet.
  3. Order of sample collection: 1) EDTA plasma, 2) SST serum, 3) other tube types.
  4. Tubes with anti-coagulants e.g. EDTA and heparin, need to be inverted 8 -10 times and placed in the rack in an upright position.
  5. Keep the samples at room temperature if processed within 1 h. Place them on ice or refrigerator if delay for more than 1 h. The samples must be processed within 4 h of collection.

**C Urine**

1. Samples collection method: Clean catch first morning void. Patients should be provided with instructions on how to collect a clean catch urine sample and provided with a sterile specimen container and a leak proof cap.
2. Samples should be stored on wet ice immediately. If collected at home, samples should be kept refrigerated and delivered in an ice pack to the clinic (at 4°C).
3. Fasting status at time of collection: Fasted since: __: __ hrs. (except plain water).
4. Record whether First Morning Void or Spot Urine. Any urination during the night if first morning void.

**D Saliva**

1. Samples collection method: Record whether patient fasted and the duration of fast (at least 6 h).
2. Record recent exposure to toothpaste, gum, cigarettes, alcohol, meal, medications (in the last 24 h). Minimum criteria for collection: 1 h after brushing teeth, at least 1 h after eating a meal, at least 12 h after last alcohol consumption, at least 20 min after last acidic food consumption e.g. citrus fruits / high sugar food).
3. Patient should rinse mouth before collection.
4. Collect sample without spitting, by drooling into the container. Showing mouth-watering images such as lemons can increase saliva production. If for DNA, use manufacturer’s protocol.
5. Obtain 2 ml of saliva apart from foam / bubbles.

**E Peritoneal fluid**

1. Samples collection method: after premedication or anaesthesia, using 20 mls suction device, laparoscopic needle and manual aspiration using syringe.
2. If small volume of peritoneal fluid / no fluid, then wash the pelvis with 20 ml sterile normal saline solution using laparoscopic needle under direct visual control.
3. Record date, time, volume and method of sample collection.
4. Transfer sample in screw top vial on wet ice within 30 min.

**F Endometrial fluid**

1. Samples collection method:
2. Before premedication or anaesthesia
3. Aspiration method using embryo-transfer catheter or cornier pipelle
4. Lavage method – Infuse 4 ml of saline into uterine cavity slowly and slowly withdraw.
5. Record date, time, volume and method of sample collection.
6. Insert the tip of the catheter into a 1.5 ml Eppendorf tube
7. Transfer sample in screw top vial on crushed ice as soon as possible.

**2 Sample Processing:**

- Pre label aliquots with identifier number followed by unique aliquot ID number, date and time of sample creation. Record type, number and volume of aliquots prepared.
- Record time of starting sample processing on log sheet.
- Store aliquots in liquid nitrogen freezers or -80°c or colder freezers,
- Any SOP deviations, problems should be recorded.
- A detailed sample freezer log should be maintained

**A Tissue**

1. Visual inspection of tissue size should be made and tissue dissected to appropriately sized pieces. Collect in the following order: 1) snap freezing, 2) RNA stabilising solution followed by freezing, 3) universal molecular fixative or formalin fixation. Please note: If time to freezing is likely to be more than 5 min, then consider immersion into RNA stabiliser as first priority.
2. Fresh tissues: Fresh tissue can be stored in fluid (e.g. media or HEPES-buffered media containing antibiotics and fungicide for incubations carried out outside a gassed incubator) up to 24 h at 4°C. Transfer to waterbath (37°C) to keep cells alive for cell culture.
3. Frozen Tissues**:** Tissue samples should be stored in an aliquot vial with screw top gasket closure. Snap freeze in liquid nitrogen as soon as possible, record time, and transfer to freezers for long-term storage
4. RNA stabilizing solution: commercially available products like Allprotect Tissue Reagent, DNA/RNA shield, PAXgene tissue containers and RNAlater are all useful as tissue RNA stabilisers. Before immersion in RNA stabilizing solution, cut large tissue samples to size according to manufacturer’s protocol. Tissue samples should be stored in an aliquot vial with screw top gasket closure containing RNA stabilizing solution within 15 min of collection, and stored at 4°C for 24 h prior to freezing.
5. Fixed tissues: Tissue samples should be mounted flat in a cassette into 20ml of 10% NBF, within 15 min (record time). The sample is transported at room temperature or on ice. The tissue should remain in NBF for no more than 24 h before transfer to graded alcohol solutions and paraffin embedding

**B Blood**

1. Allow SST tubes to clot for 30 min in upright position at room temperature and
2. Centrifuge samples for 10 min, at 2500 g at 4°C within 1 h of collection but store on ice if this time is exceeded.
3. Place the tubes upright on a rack and on ice during aliquotting.
4. Set number and pre label aliquot tubes, aliquot into small samples of 100 – 500μl to minimize the freeze thaw cycles.
5. Aspirate and transfer all the plasma or serum using a pipette carefully not disturbing the cell layer below, holding the tube at 45° angle, into a vial with screw top gasket closure. If there is contamination with cells then the vials can be re-centrifuged and transferred to new vials.
6. White blood cell (WBC) aliquots: Aspirate and transfer the buffy coat layer from the collection tube into a vial.
7. Red blood cell (RBC) aliquots: Gently mix and transfer the remaining erythrocytes into another vial.
8. Record volumes of plasma/serum, WBC, RBC in each aliquot.
9. Samples should be processed and stored within 1 h of collection

**C Urine**

1. All samples should be kept refrigerated and processed within 2h of collection.
2. Discard samples if they contain blood and record it.
3. Mix sample by swirling or repeated pipetting.
4. Dipstick analysis: retest if specific gravity <1.001 or >1.032, record all the findings
5. Store the unprocessed urine aliquots in LN_2_ or in an -80^o^C or colder freezer.
6. Fill sterile tube with remaining urine and centrifuge at 1000-3000 g at 4^o^C for 5 min.
7. Place samples on wet ice and aspirate supernatant into aliquots. Label aliquots as above (in 5 and store processed urine as in 6).
8. Samples should be processed and stored within 2h

**D Saliva**

1. Samples should be stored on wet ice or refrigerator if more than one hour for processing.
2. Aliquot the necessary amount into vials for processing, remaining unprocessed saliva store in a screw top vial with gasket closure, label them and store in liquid nitrogen or - 80°C / lower freezers
3. Centrifuge saliva (to be processed) at 1000 g at 4^o^C for 2 min.
4. Aspirate supernatant into aliquots.
5. If for RNA extraction: Aliquot the centrifuged sample into a vial with RNA stabiliser (RNA Protect saliva kit by Qiagen). Label the aliquots consistent with initial sample label and store.
6. Saliva samples should be processed and stored within 4h

**E Peritoneal/ Ascitic fluid**

1. Record colour, clarity and volume of the sample
2. Centrifuge sample at 900 g at 4^o^C for 5 min.
3. Aspirate supernatant into aliquots with a screw top gasket closure and fill as close to the surface as possible. Transfer the pellet to an appropriate sized aliquot vial and stored.
4. Store fluid aliquots in liquid nitrogen freezers or -80°c or colder freezers

**F Endometrial fluid / aspirate**

1. If aspirate larger than 700ul, homogenise and split into two tubes to make two equal aliquots.
2. Add 1:1 ratio of PBS to the sample and shake manually by inverting the tube several times.
3. Centrifuge sample at 2500 g at 4^o^C for 20 min.
4. Aspirate supernatant into a new labelled eppendorf. Store the eppendorf tube with the pellet.
5. Store both fluid aliquots in liquid nitrogen freezers or -80°c or colder freezers

**3. Storage and data recording**

- Date and time of storage should be recorded on the log sheet. Also record the type, number of aliquots prepared on the biospecimen form. Store samples in LN_2_ freezers (if available), as they have less temperature fluctuations than -80°C freezers.
- Record on the log sheet any variations or deviations from the SOP; also document if any problems, or issues (e.g. vial cracked during processing).
- Record the location of each sample into the freezer including freezer number, rack, box, and spot in the box along with all other sample attributes in a database.
- Keep a record of any freeze-thaw events that occurs with a sample for any reason.
- Track any change of location of a sample, including sending a sample out to an assay lab for processing.
- Track any new samples created from the original sample (i.e. a sub-sample) in the same manner as described above. Ensure that each sub-sample/aliquot is labelled with a unique ID.

**4. Freezer check:**

- Installing a centralized freezer/liquid nitrogen-monitoring system automatically records temperature fluctuations and makes call-outs to responsible persons in the event of a problem. If not, Check freezers twice weekly and keep a written-log of checks. Have alarm systems setup on all freezers in addition to human twice-weekly checks.
- Aliquots of the same sample type and patient should be split between freezers where possible to ensure sample integrity in case of a freezer breakdown.
- Facilities should have an emergency -80°C freezer for the transfer of samples in the event of freezer breakdown.

**5. Data recording checklist**

1. Record protocols, specifying which steps are followed.
2. For each sample, record:
   1. Date and time of sample collection (Date: __/__/__ and __: __ hrs.).
   2. Start time of sample processing in the laboratory (Date: __/__/__ and __: __hrs.).
   3. Record type, number, volume of aliquots prepared
   4. Record fasting time or whether less than 1) 1 h after brushing teeth, 2) 1 h after eating a meal, 3) 12 h after last alcohol consumption, 4) 20 min after consuming acidic foods like citrus fruits/ high sugar foods – if applicable
   5. Date and time sample is stored into freezer

(Date: __/__/__ and __: __hrs.).

- 1. Any variations or deviations from the SOP, problems, or issues.
  2. Any freeze-thaw event that occurs with a sample for any reason.

1. Keep a log of twice weekly freezer checks.
