# Supplementary material for: Harmonisation of biobanking standards in endometrial cancer research
Source: Br J Cancer. 2017 Jun 29;117(4):485–93. doi: 10.1038/bjc.2017.194 (PMC5558683; doi:10.1038/bjc.2017.194)
Supplement: Supplementary Figure 1 [file bjc2017194x6.docx]

100.00%


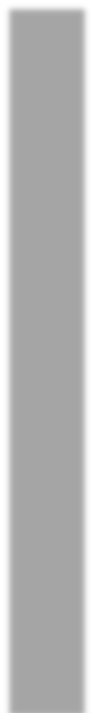

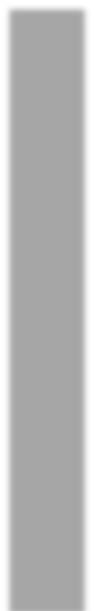

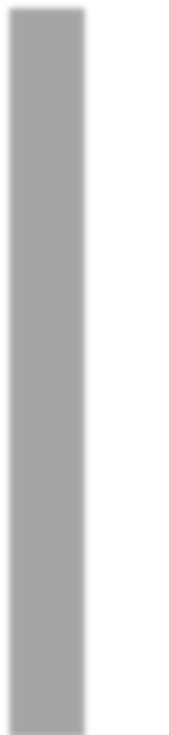

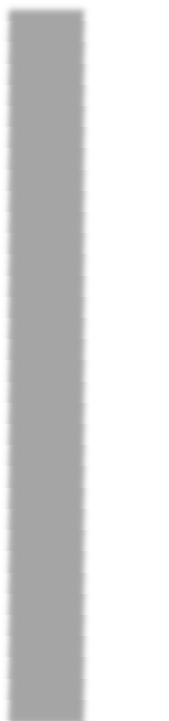

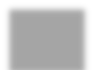

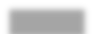

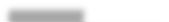

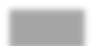

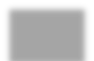

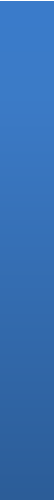

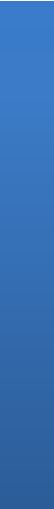

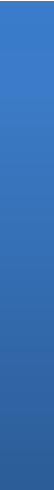

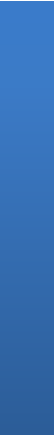

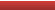

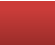

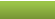

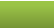

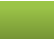


96.25%

98%

94%

83.75%

8.75%

7.50%

3.75%

6%

0

2%

0

0

90.00%

80.00%

70.00%

60.00%

50.00%

40.00%

30.00%

20.00%

10.00%

0.00%

GO PT BS PATH


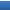

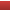

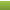
AGREE UNDECIDED DISAGREE

Figure 3. Overall percentage of scores (agree, undecided and disagree) by different panels for different tools using Modified Delphi technique.
