# Supplementary material for: Harmonisation of biobanking standards in endometrial cancer research
Source: Br J Cancer. 2017 Jun 29;117(4):485–93. doi: 10.1038/bjc.2017.194 (PMC5558683; doi:10.1038/bjc.2017.194)
Supplement: Supplementary Document Legends [file bjc2017194x7.docx]

**Supplementary information:**

1. Endometrial Cancer Patient Data (ECPD) Collection Tool
2. Endometrial Cancer Surgical Data (ECSD) Collection Tool – Standard
3. Endometrial Cancer Surgical Data (ECSD) Collection Tool – Minimal
4. Endometrial Cancer Biospecimen (ECBS) Tool
5. **Standard Operating Procedures for collection, processing and storage of the tissue, blood, urine, saliva and fluid samples for endometrial cancer research (SOP-ECBS)**
6. **Supplementary Figure 1. Overall percentage of scores (agree, undecided and disagree) by different panels for different tools using Modified Delphi technique.**
